# Statins in Chronic Kidney Disease—Effects on Atherosclerosis and Cellular Senescence

**DOI:** 10.3390/cells12131679

**Published:** 2023-06-21

**Authors:** Piotr Fularski, Julia Krzemińska, Natalia Lewandowska, Ewelina Młynarska, Maciej Saar, Magdalena Wronka, Jacek Rysz, Beata Franczyk

**Affiliations:** 1 Department of Nephrocardiology, Medical University of Lodz, ul. Zeromskiego 113, 90-549 Lodz, Poland; piotr.fularski@stud.umed.lodz.pl (P.F.); julia.krzeminska1@gmail.com (J.K.); natalia.lewandowska1@stud.umed.lodz.pl (N.L.); maciej.saar@stud.umed.lodz.pl (M.S.); wronkam96@gmail.com (M.W.); bfranczyk-skora@wp.pl (B.F.); 2 Department of Nephrology, Hypertension and Family Medicine, Medical University of Lodz, ul. Zeromskiego 113, 90-549 Lodz, Poland; jacek.rysz@umed.lodz.pl

**Keywords:** atherosclerosis, senescence, chronic kidney disease, inflammation, statins

## Abstract

Chronic kidney disease (CKD) is a serious health problem that can affect various systems in the human body. Renal failure promotes mechanisms of premature cellular aging and also features of generalized inflammation in the body, which translates into a close relationship between kidney dysfunction and cardiovascular disease (CVD). As kidney function deteriorates, cardiovascular risk and mortality increase in this group of patients. Oxidative stress and inflammation are two closely related processes that initiate a vicious cycle by activating each other. Together with aging, they represent the key factors that cause and exacerbate CVD in CKD. Patients with CKD are particularly vulnerable to the accumulation of aging endothelial cells, vascular smooth muscle and macrophages, increasing the risk of atherosclerosis. Several mechanisms are known that can lead to the progression of the aforementioned problems, such as the accumulation of uremic toxins, persistent inflammation, impaired lipid and electrolyte metabolism, nitric oxide (NO) deficiency, the increased production of reactive oxygen species (ROS) and damage to deoxyribonucleic acid (DNA) and mitochondria. According to research, we can distinguish a group of drugs that effectively counteract the negative effects of CKD—statins. This is a group of drugs that inhibit 3-hydroxy-3-methylglutaryl-coenzyme-A (HMG-CoA) reductase and affect a number of cellular processes and pathways, resulting in the overall slowing of atherosclerosis and cellular aging.

## 1. Introduction

One of the hallmarks of aging is cellular aging. This is a process in which a cell stably exits the cell cycle as a result of cellular damage or stress [1]. When senescent cells permanently stop their growth, there are also profound changes in their secretory phenotypes. Along with aging and injury, they accumulate in many organs, including the kidneys, which contributes to the development of age-related diseases and thus to limiting a person’s life, during which he is generally in good health. Recently, there has been increasing interest in the process of cellular senescence. New strategies to manipulate this process are constantly being sought to discover a new therapeutic target in kidney disease [2,3]. The most attractive proposals are related to the attenuation of the secretory phenotype associated with senescence (SASP) or the elimination of senescent cells, which are supposed to contribute to the delay of the onset of age-related diseases or even their reversal [4].

Oxidative–antioxidant imbalance leads to oxidative stress [5]. Combined with inflammation, this process plays an important role in aging [6,7,8,9,10], accelerates the process of aging [11] and increases the risk of developing diseases [10]. Oxidative stress underlies many chronic or degenerative diseases, such as diabetes, cardiovascular disease (CVD) [12,13], cancer [14], lung disease, skeletal muscle disease [7], acute and chronic kidney disease (CKD), age-related macular degeneration, neurodegenerative diseases [6] and others. Table 1 lists age-related diseases with their SASP profiles [6].

Studies emphasize the role of oxidative stress and inflammation in vascular aging, which underlies many age-related diseases [6,15]. Reactive oxygen species (ROS) overactivity reduces the vasodilatory capacity of NO. The activation of pro-inflammatory nuclear factor-κB (NF-κB) additionally enhances oxidative stress [15]. Meanwhile, the antioxidant response is reduced by decreased levels of glutathione peroxidase, superoxide dismutase (SOD) [6], mitochondrial manganese superoxide dismutase (SOD2) and nuclear factor erythroid 2-related factor 2 (Nrf2) [15], which negatively affects the heart’s tolerance to oxidative stress [6]. This series of processes leads to endothelial dysfunction and ultimately to the development of a number of CVDs [15].

Oxidative stress and inflammation influence the formation of CKD. The increased formation of ROS due to the activation of polymorphonuclear leukocytes and monocytes, as well as increased homocysteine levels, is observed. The formation of arterial hypertension in the course of CKD is enhanced by reduced nitric oxide (NO) concentrations caused by O_2_^·−^ and high concentrations of asymmetric dimethyl arginine (ADMA) [6].

Aging processes cause the accumulation of aging endothelial cells, vascular smooth muscle cells (VSMCs) and macrophages, which, leading to chronic inflammation, increase the risk of atherosclerosis [16,17]. Its development may be affected by a variety of risk factors, such as lipid level disturbances, which often occur in patients with CKD [18]. Atherosclerosis also has a negative impact on the antioxidant ability of the organism, resulting in increased oxidation, which further contributes to vascular dysfunction and calcification of the vessels [19]. Cellular aging processes and vascular changes such as atherosclerosis in the course of CKD are caused by, among other factors, the accumulation of uremic toxins, phosphate retention or the reduced expression of the Klotho protein [20,21]. These factors consequently contribute to oxidative stress and inflammation, and subsequently induce damage to mitochondria and deoxyribonucleic acid (DNA) and telomere shortening [21]. It is suspected that actions to remove aging cells may protect against the formation of atherosclerosis [22].

CKD is an example of premature aging, in which early vascular aging and calcification are particularly prominent, which can eventually lead to chronic vascular disease [23,24]. Chronic inflammation is characteristic of aging processes, and the formation of a pro-inflammatory environment in the walls of blood vessels predisposes patients to vascular dysfunction and the formation of vascular disease. Moreover, oxidative stress plays a major role in the development of age-related vascular pathological changes, including arterial stiffness or endothelial dysfunction [22]. Focusing on the early process of vascular aging in CKD is important because it ultimately increases the risk of cardiovascular mortality [23,24].

## 2. Development of Atherosclerosis in Chronic Kidney Disease

Atherosclerosis is a severe health problem concerning the cardiovascular system. It is based on the aggregation of specific elements among the arteries, such as cholesterol, fibrous components and calcium. This further promotes a decrease in vessel diameter and local inflammation, resulting in the development of atherosclerotic plaques [25].

CKD is linked with the advancement of atherosclerosis and a higher probability of cardiac incidents [26]. Patients with CKD exhibit a higher probability of coronary occlusions and mortality, which is correlated with raised concentrations of inflammation-related markers such as interleukin-6 (IL-6), C-reactive protein and tumor necrosis factor [27]. Peripheral artery disease (PAD), which is a result of the atherosclerotic process, is also more prevalent in the group of patients with an estimated glomerular filtration rate (eGFR) at the level of 60 mL/min/1.73 m^2^ or less [28]. There are a few known mechanisms responsible for rapid aging in this specific disease, which further contributes to atheromatous plaque growth. CKD causes the increased deterioration of biological molecules and a deficiency in antioxidant defense mechanisms, which implies the reduced elimination of ROS and its higher amounts in the body. CKD is also characterized by elevated levels of uremic toxins in the bloodstream. Moreover, different essential processes that represent biological senescence, such as the impaired functioning of mitochondria, cellular aging, modified interactions between cells or the disruption of proteostasis, can also play crucial roles in the development of CVD related to CKD [24]. Interestingly, patients with CKD present a particular lipid profile called “uremic dyslipidemia” that is marked by diminished high-density lipoprotein cholesterol, low-density lipoprotein cholesterol (LDLc) within the norm and elevated triglyceride levels in serum. There are also modifications in the configuration and molecular arrangement of lipoproteins. This is a result of oxidation, carbamylation, the reduced clearance or accumulation of small particles, modified metabolism, a changed proteome and an abnormal lipidome, which directly affect the biological functions of lipoproteins [29]. Moreover, it has been observed that the homocysteine quantity is significantly raised in this patient group. This implies the aggravation of LDLc oxidation and promotion of atherosclerosis within the kidneys. Furthermore, they also have elevated phosphate levels, which, after binding with calcium, may initiate inflammatory reactions in macrophages. In general, the inflammatory environment in this disease is mainly considered to be a result of the overproduction and lowered removal of pro-inflammatory agents [30]. Increasing sources are revealing that there is a strong correlation between atheromatous plaque formation in both marginal and cardiac arteries and the phosphate level. The mechanism by which atherosclerosis is induced by the phosphate level is not understood yet. It is suggested that this can be attributed to the disruption of endothelium functioning, which further leads to lipid accumulation within the arteries and promotes atherosclerosis. Nevertheless, further randomized studies on large patient groups should be performed to explain this issue more precisely [31].

In addition, leptin is one of the substances that shows elevated levels in CKD. Leptin is considered an agent with pro-atherosclerotic abilities due to its aggravation of persistent inflammation and intensification of oxidative stress. It also shows an effect on the advancement of CKD via a hypertensive influence. The correlation between leptin and the risk of cardiovascular events and its severity is still being examined. Authors suggest that more studies need to be performed on leptin’s contribution to the development of atheromatosis [32]. 

**Figure 1:**
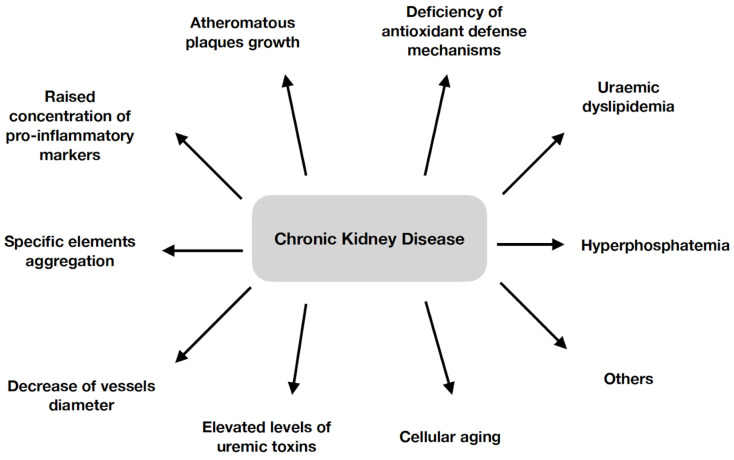
Effects of CKD on atherosclerosis [20,24,25,27,29,30].

A few examples of how CKD affects atherosclerosis are shown in Figure 1 [20,24,25,27,29,30]. 

Osteopontin and fetuin A are two notable markers in terms of atherosclerosis. The former plays a significant role in terms of providing information on whether the calcification process has already been initiated. On the other hand, it is unable to serve as a predictor of progression. The level of fetuin A can be used to assess the risk of atherosclerosis in a group of patients with CKD, since its levels begin to decline at the onset of the disease and are significantly reduced in patients suffering from atheromatosis [33,34]. During our research, we have not identified any significant contradictions or a lack of information on the specific topics.

## 3. Statins in Chronic Kidney Disease

CKD poses a serious threat to the health and well-being of numerous patients around the world [35]. It is also important to mention that a constant state of inflammation is proven to lead to the aggravation of the clinical state of the patient [36].

**Figure 2:**
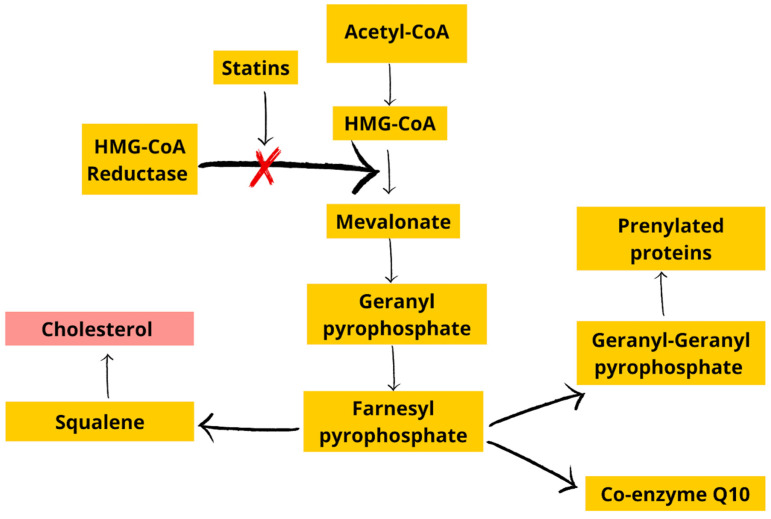
The mevalonate pathway [40]. Acetyl-CoA, acetyl-coenzyme-A; HMG-CoA, 3-hydroxy-3-methylglutaryl-coenzyme-A.

Statins act as inhibitors of 3-hydroxy-3-methylglutaryl-coenzyme-A (HMG-CoA) reductase, inhibiting an important enzymatic pathway that leads to cholesterol production (Figure 2). Statins are a type of lipid-lowering medication used widely in patients with a high risk of CVD [37]. These medications (mevastatin, lovastatin, pravastatin) are fully derived from natural sources, such as fungi including *Aspergillus* and *Penicillium* or fermented red rice [38]. Thanks to their ability to lower mortality rates and the severity of illness, these substances are frequently used as cholesterol-reducing medicines [39].

HMG-CoA reductase inhibitors (statins) might exert a beneficial effect through several mechanisms. By inhibiting HMG-CoA reductase, statins lower the levels of cholesterol and also decrease the amounts of intermediate products of cholesterol synthesis, which influence intracellular signaling, cytokine expression, chemokine regulation, reductions in adhesion molecule expression in leukocytes and endothelial cells, the modulations of the immunological system and anticoagulant effects [39]. Dyslipidemia has been shown to be associated with CKD [41]. In addition, serum lipoprotein A and C-reactive protein levels are elevated, so statins are used for treatment [42]. Although LDLc levels are not strictly associated with clinical deterioration, elevated triglyceride levels are proven to be detrimental [43]. Therefore, disease progression is possible. It is noteworthy that there are meta-analyses that show a positive effect of statins on CKD progression at steady state [44,45]. According to Kidney Disease Improving Global Outcomes (KDIGO), statins should be added to the therapy in patients with a high cardiovascular risk due to dyslipidemia who have an estimated glomerular filtration rate of less than 60 mL/min/1.73 m^2^, with the exception of hemodialysis patients [46]. 

Using the data acquired from the meta-analysis by Esmeijer et al. [47], it was determined that statins have a beneficial influence on patients with CKD. The effects of statins were divided into two categories, where the strongest influence on eGFR decline was exerted by the combination of fluvastatin 20 mg and ezetimibe 10 mg; the next was the combination of rosuvastatin 20 mg with ezetimibe 10 mg, followed by single pravastatin 10–20 mg and atorvastatin 40–80 mg. The most visible changes regarding proteinuria for the benefit of the patient were achieved by the combination of fluvastatin 20 mg/ezetimibe 10 mg, the combination of of rosuvastatin 20 mg and ezetimibe 10 mg and lastly the monotherapy of atorvastatin 40–80 mg [47].

Of note is that the surface under the cumulative ranking curve (SUCRA) provides information about the effectiveness of a method/technology. The higher value, which is stated as a percentage, the greater the likelihood of success for the mentioned method or therapy [47].

Regarding the eGFR SUCRA value, it was estimated to be the highest for the combination of fluvastatin 20 mg and ezetimibe 10 mg (99%), whereas the combination of fluvastatin 20 mg and ezetimibe 10 mg (86%) yielded the highest SUCRA value for the alteration of proteinuria, but of note is also the high efficacy of the monotherapy of atorvastatin 40–80 mg (78%) [47].

## 4. Senescence

Organismal aging is a complex process that consists of many factors. It is characterized by the progressive deterioration of various systems and biological functions in the body, which leads to a decrease in overall health and increased susceptibility to disease. One of the most important characteristics of aging is cellular senescence. This is a stress response that causes permanent cell cycle arrest, and senescent cells lose their ability to proliferate, even in the presence of mitogenic stimuli. This phenomenon can occur in all types of somatic cells through the actions of different stimuli [1,4,48]. Cellular senescence can be induced by diverse stress stimuli, which can be divided into extrinsic and intrinsic factors. Internal factors include replicative stress caused by telomere shortening and the induction of a DNA damage response, oncogene activation, epigenetic alterations, genomic instability, deficient DNA repair, reactive metabolites or oxidative stress. Meanwhile, therapy-induced stress caused by chemotherapeutics and radiation or UV light, chemical mutagens and viral infections belong to external factors [1]. Senescent cells have different morphological and molecular features and functions from other cells that have also lost the ability to divide. These include prolonged and generally irreversible cell cycle arrest, the secretion of a range of pro-inflammatory and proteolytic factors as part of the SASP, macromolecular damage, deregulated metabolism and transcriptional changes [49]. Cellular senescence is an essential biological process that contributes to the reduction of tumorigenesis and lifelong tissue damage and is involved in morphogenesis, regeneration and wound healing. However, lingering senescent cells that increasingly accumulate in organs drive age-related disorders. Cells remain metabolically active and secrete many senescence-associated factors [4]. SASP and its components are capable of promoting chronic inflammation and tissue dysfunction, resulting in the loss of their repair and regeneration. These components include, among others, pro-inflammatory cytokines, chemokines, metabolites, proteases, bioactive lipids, inhibitory molecules and extracellular vesicles [1,50]. The immune system is chronically activated in response to these factors, resulting in reduced senescent cell clearance. This creates a vicious cycle that further fuels inflammation and the negative effects of cellular senescence [51].

The results of preclinical research have shown that many disorders can be ameliorated, delayed and even prevented by interventions involving senescent cells that lead to pathological tissue damage [52]. These results, and the emerging opportunity to delay aging and the development of other diseases, have sparked interest in “senotherapy”, which targets senescent cells [53]. A variety of therapeutic options have been developed that pharmacologically eliminate senescent cells, called senolytics, or lead to the suppression of SASP and other markers of senescence, known as senomorphics [1]. Some of these senotherapeutics are already at the stage of clinical trials and their use in humans brings hope for the future treatment of age-related diseases and dysfunction, such as atherosclerosis, cancer, diabetes, neurodegenerative diseases, CVD and CKD [51].

Using vascular cells as an example, it has been shown that cellular senescence can be triggered by chronic inflammation. This ultimately leads to endothelial dysfunction, which promotes atherosclerosis [54] and other CVDs [55].

**Figure 3:**
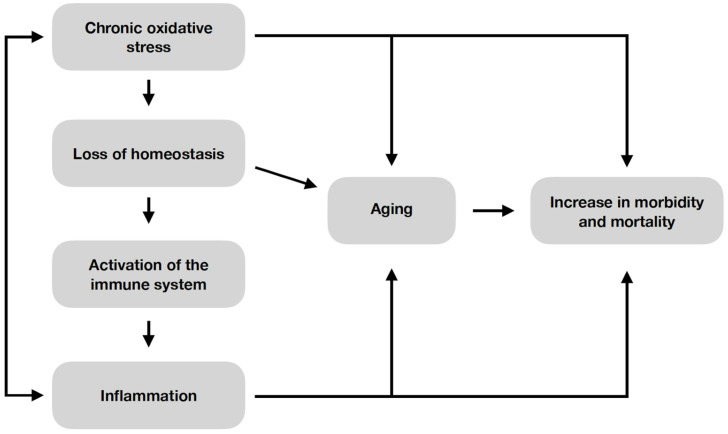
The “oxi-inflamm-aging hypothesis” [6].

According to the “oxi-inflamm-aging hypothesis”, excessive reactive oxygen and nitrogen species (RONS) are the basis of aging-related processes. According to this theory, oxidative stress causes the activation of, among others, the immune system. The result is inflammation. A crucial part of the aging process is cellular senescence, which is based on the inhibition of cellular proliferation. This process is caused by damage to proteins, lipids and nucleic acids caused by the accumulation of RONS. The “oxi-inflamm-aging hypothesis” is visualized in Figure 3 [6].

Another important link between aging and oxidative stress is the role of Nrf2 signaling [8,9]. Nrf2 signaling is involved in many molecular mechanisms associated with aging, and its activity decreases with age. In contrast, Nrf2′s antioxidant properties are mainly based on its regulation of enzymes such as catalase, SOD and heme oxygenase-1, among others. However, the overexpression of Nrf2 may be associated with negative consequences for the organism, such as malignant transformation [9]. Zhang et al. [8] report that a decrease in Nrf2/electrophile-responsive element signaling with advancing age results in a decline in the body’s ability to respond properly to ROS accumulation. Coinciding with these findings are reports by Yu et al. [9] showing a Kelch-like enoyl-CoA hydratase-associated protein 1 (Keap1)–Nrf2 system. Due to its regulatory properties against antioxidant enzymes, Keap1–Nrf2 can be used to observe the level of oxidative stress. It is also considered an important system to protect the cell from ROS accumulation [9]. However, the authors of the study emphasize that a thorough understanding of the Nrf2 signaling pathways in aging still requires much research and analysis [8,9]. 

## 5. Correlation between Cellular Senescence Process and Atherosclerosis in Chronic Kidney Disease

CKD is one of the most exemplary and common diseases that promote premature cellular aging, as inferred from the numerous pathological phenomena and conditions that accompany this disease [21,24]. Renal failure promotes not only mechanisms of premature aging but also features of generalized inflammation, which translates into a close relationship between renal dysfunction and CVD [24,56]. A number of unfavorable mechanisms leading to premature aging in CKD can be listed, among which are active systems that accelerate aging, the disruption of anti-aging systems, generalized inflammation and increased oxidative stress [23]. Oxidative stress and inflammation are two closely related processes that trigger a vicious cycle by activating each other. Together with aging, they are the key factors that cause and exacerbate CVD in CKD [24]. The inflammatory process in CKD not only results from aging but also initiates it [23].

With age, vessels are switched to a pro-inflammatory, pro-coagulant direction and are prone to narrowing [17]. CKD patients are burdened by two types of arterial disease, atherosclerosis and arteriosclerosis, which do not always occur together [56]. Arteriosclerosis is primarily associated with systemic inflammation and is primarily caused by two components—lipid disorders and endothelial dysfunction [24,57]. CKD predisposes patients to both, increasing the risk of atherosclerosis [57]. Atherosclerotic lesions contain increased numbers of aging, apoptotic cells, including endothelial and foam cells and VSMCs with increased tumor protein 53 (p53) expression [17,22,57]. These cells exhibit growth arrest and an enlarged and flattened phenotype and likely contribute to the instability of atherosclerotic plaques by, among others, exacerbating inflammation [22,57]. It has also been suggested that the development of atherosclerosis may be favored by a deficiency in progenitor cells [22]. Importantly, patients with CKD are characterized by more calcified atherosclerotic plaques, unstable with less fibrous tissue [58]. Aging cells secrete a number of factors, such as pro-inflammatory cytokines and chemokines, that increase inflammation and promote cell growth arrest, creating a vicious cycle, and this process is characteristic of the development of atherosclerosis [21]. It has been found that the vascular damage and remodeling characteristic of CKD is caused by the aging of both vascular cells and peripheral blood cells [24].

It has been shown that in the course of CKD, endothelial cells are aged by homocysteine, indoxyl sulfate or advanced glycation end products, which are uremic toxins. Progressive renal dysfunction increases the amount of circulating uremic toxins, which in turn promotes oxidative stress and inflammation [24]. Renal dysfunction and uremia, in the same way, through pro-inflammatory factors, lead to aging and impair the differentiation of pluripotent stem cells. As a result, the number of atypical and pro-inflammatory monocytes increases, and the number of endothelial cells with repair potential decreases, leading to endothelial damage [17,21]. Urea decreases the availability of NO necessary for vasodilation by producing increased amounts of ADMA, which inhibits NO synthase and consequently promotes endothelial dysfunction [56]. IS also plays a major role, inhibiting endothelial nitric oxide synthase (eNOS), increasing the levels of ROS, causing endothelial dysfunction and decreasing the protective potential through, among other factors, signaling by Nrf2. All of this also results in increased levels of pro-inflammatory cytokines [24]. Increased DNA damage also plays a significant role in vascular aging—vascular endothelial calcification and arterial stiffness. In the course of CKD, uremic toxins disrupt the protective activity of systems that counteract DNA damage promoting aging, which induces vascular wall calcification. Moreover, aging VSMCs secrete some of the SASPs, such as IL-6 and osteoprotegerin, which promote both local and distant calcification. There is evidence that uremic toxins also contribute to endothelial calcification by differentiating VSMCs into osteochondrocytes, inducing arterial stiffness [23]. Reduced mitochondrial function, which overproduces ROS, is also characteristic of atherosclerosis development in uremia [21,22]. They activate NF-kB pathways, matrix metalloproteinases (MMPs) and the B-cell lymphoma (Bcl-2)-dependent pathway to induce apoptosis, promoting vascular inflammation and aging. Mitochondria are suspected to contribute to aging processes as a result of mutations in mitochondrial DNA, which consequently disrupts their energy production, and such changes have been detected in atherosclerotic plaques [22]. It has been suggested that aging may also be promoted by the mutation of the nuclear plaque lamina A protein. Indoxyl sulfate, contained in uremic toxins, interferes with the conversion of prelamin A to its mature form, resulting in its accumulation in VSMCs. The consequence of these processes is DNA damage, the premature aging of VSMCs and calcification [23].

CKD leads to the dysregulation of the immune system and is characterized by numerous changes in the immune system, both morphologically and functionally in its components [23,24]. The correlation between immune system cellular aging and premature CVD is noteworthy [21,24]. In renal failure, the subpopulation of tissue-aggressive clusters of differentiation (CD)14+ and CD16+ monocytes increases, which can be considered a sign of generalized aging [24,56,59]. Intermediate monocytes (CD14++/CD16+) and non-classical monocytes (CD14+/CD16+) have been shown to have the characteristics of activated cells, exhibit a pro-inflammatory nature and may contribute to atherosclerosis [24,59]. In particular, non-classical monocytes have a high pro-inflammatory capacity, and they are the ones that accumulate in people with CKD. Not only are they highly resistant to apoptosis, but they also have a strong capacity to adhere to endothelial cells by accumulating many adhesion receptors, such as vascular cell adhesion molecule 1 (VCAM-1) or intercellular adhesion molecule 1 (ICAM-1), where they then promote vascular damage and contribute to CVD [24,59]. One study showed that among a subpopulation of aggressive monocytes in CKD, the telomere length was shorter, which is associated with a higher risk of patient death [56]. All of this implies that, along with generalized inflammation, these cells may play an important role in the pathogenesis of atherosclerosis, and their number is directly proportional to endothelial damage [59].

Anti-aging systems include fetuin-A and the Klotho protein [23]. The Klotho protein is considered to be closely related to renal dysfunction, since its levels are reduced in CKD, and its absence is associated with the aging phenotype and arterial stiffness [21,23]. Through increased phosphate excretion, it prevents hyperphosphatemia and consequently vascular calcification and the apoptosis of VSMCs. Table 2 lists some of the anti-aging characteristics of the Klotho protein [21]. In contrast, low levels of the glycoprotein fetuin-A, whose function is to bind calcium and prevent calcification, co-occur with telomere shortening. Unfortunately, the function of both systems in CKD is impaired by hypercalcemia, hyperphosphatemia or hyperparathyroidism [23].

Several researchers have focused on the link between the propensity for atherosclerosis and arterial calcification and the incidence of CKD [58,60,61]. A meta-analysis by Ho et al. [60] assessed the prevalence of PAD in patients with CKD in Australia. They found that PAD was significantly more common in patients with end-stage kidney disease than in non-dialysis patients; moreover, older, Caucasian hemodialysis patients were more predisposed to PAD [60]. McCullough et al. [61] also focused on CKD patients with vascular calcification and similarly showed that elderly dialysis patients with dyslipidemic features were particularly predisposed to vascular calcification. However, they did not show a relationship between the degree of calcium and phosphate burden, their treatment and vascular calcification in this group of patients [61]. On the other hand, Kousios et al. [58] evaluated the relationship between the levels of MMPs, tissue inhibitors and arteriosclerosis in patients with CKD. The carotid intima–media thickness was evaluated as an indicator of arteriosclerosis. MMP2 was shown to be the only one that correlated with the carotid intima–media membrane thickness, demonstrating that MMPs may be a useful biomarker to indicate signs of subclinical atherosclerosis, which is a predictor of CVD [16,22]. This relationship can be seen, among others, in a mouse model, in which the elimination of aging cells led to the regression of atherosclerotic plaques. In the same way, the induction of the apoptosis of aging cells may contribute to the reduction of vascular ossification and aortic calcification [16].

## 6. Effects of the Use of Statins on the Process of Cellular Senescence

Statins are drugs with pleiotropic effects [55,62]. By inhibiting HMG-CoA reductase, they affect vascular and nonvascular tissues. In addition to lowering LDLc cholesterol, they have been shown to have beneficial effects on endothelial progenitor cells (EPCs) and the vascular endothelium by improving NO availability [55]. Statins, by activating the phosphatidylinositol 3-kinases/protein kinase B (PI3K/Akt) pathway [63], modulate gene expression, affecting, among other aspects, the cell cycle, cell proliferation or cell aging processes [55,63]. In addition, they inhibit telomere shortening and delay senescence [62]. In vitro studies have shown that the use of statins causes EPCs to increase in number, have better viability and, most importantly for this review, have fewer senescence characteristics [55]. Similar conclusions were obtained in a study by Assmus et al. [63], in which it was shown that under ex vivo conditions, statins increase the proliferation and prevent the cellular senescence of EPCs, in which the geranylgeranyl pathway is involved. The inhibition of cellular senescence in a dose-dependent manner has been observed for both atorvastatin (the most effective dose is 0.1 μmol/L) and mevastatin [63].

Another HMG-CoA reductase inhibitor, pravastatin, was reported in a study on human lung fibroblasts by Ushijima et al. [64]. The aim of the experiment was to find a drug that reduced the viability of senescent lung fibroblasts. For this purpose, several groups of drugs and substances were tested and the viability of the treated cells was measured using the Cell Counting Kit 8 assay. It was shown that in vitro pravastatin selectively inhibited senescent cell proliferation [64]. Notably, senescent fibroblasts can promote tumorigenesis through SASP [65]. A study by Liu et al. [65] showed that simvastatin suppresses SASP by inhibiting protein prenylation and inhibits Ras-related C3 botulinum toxin substrate (Rac1) and cell division control protein 42 analog (Cdc42) activation. This suggests that statin use attenuates cancer-promoting processes [65].

There have also been studies on the development of senescence in human dermal fibroblasts. Statins, acting on the mevalonate pathway, can inhibit the synthesis of coenzyme Q10 (CoQ10), the amount of which decreases with age, and it is associated with the aging process [66,67]. The effect of simvastatin on oxidative stress and CoQ10 content was shown to be dependent on the concentration at which cells were incubated. Higher concentrations of statin (>10,000 nM) increased oxidative stress, leading to mitochondrial dysfunction and cell death, while lower concentrations (<300 nM) induced improvements in cellular oxidative status through adaptive mechanisms. The change in cellular oxidative status and mitochondrial dysfunction induced by high doses of statins promoted premature senescence. This was evidenced by an increase in ß-gal positivity and an increase in p16 messenger ribonucleic acid and protein expression in simvastatin-treated cells compared to control cells. The lower concentrations of statins used in the study (which corresponded to standard therapeutic doses of statins in humans, i.e., 40–80 mg/day) did not result in the inhibition of CoQ10 synthesis; oxidative stress was maintained at low levels, and, most importantly, there was no increase in markers of cellular senescence [66]. In addition, further studies have shown that providing CoQ10 prevents the reduction of its levels and thus the aging processes and cellular senescence of skin fibroblasts [67]. 

Motoji et al. [54] conducted a study in mice to test how vasculitis and statins affect cellular senescence and vascular endothelial cells. The study used atorvastatin at a dose of 10 mg/kg/day. Senescence was measured via the expression of cellular senescence markers such as p53, ICAM-1, VCAM-1 and phospho-retinoblastoma protein in ribonucleic acid extracted from aortic tissue. Statin use was not shown to inhibit vascular cellular senescence induced by vascular inflammation. However, a study revealed that statins affecting the phosphatidylinositol 3-kinases/protein kinase B/endothelial nitric oxide synthase (PI3K/Akt/eNOS) and Ras homolog family member 1/rho-associated, coiled-coil-containing protein kinase 1 (Rho/ROCK) pathways increased Akt and eNOS activity, thereby improving endothelial cell function [54].

## 7. Conclusions

Uremia, in the course of chronic kidney disease, promotes many mechanisms of premature aging and may therefore be considered a promoter of this process. The accumulation of aging vascular cells, oxidative stress and chronic inflammation play a particular role in the development of CKD-related vascular dysfunction, including arterial stiffness, endothelial dysfunction, calcium deposition and atherosclerosis. Patients with CKD are particularly vulnerable to vascular damage and remodeling, and these can ultimately lead to CVD and death. It is suspected that efforts to remove aging cells may protect against the development of atherosclerosis and other vascular lesions. CKD is inextricably linked to lipid metabolism and electrolyte disturbances, uremic toxin accumulation, oxidative stress, inflammation and NO deficiency. Aging cells have been detected in atherosclerotic lesions, which likely contribute to the instability of atherosclerotic plaques, including by exacerbating inflammation. Statins, by inhibiting HMG-CoA reductase, affect multiple cellular processes and pathways, achieving a pleiotropic effect. They have been shown to inhibit senescence in a dose-dependent manner. A similar correlation has been shown for oxidative stress—low doses of statins cause improvements in cellular oxidative status, while high doses exacerbate oxidative stress, ultimately leading to cell death. Statins selectively inhibit senescent cell proliferation, and their use attenuates cancer-promoting processes. Although statins are used as important drugs against dyslipidemia, which is often present in patients with CKD, normolipidemic patients also respond positively and mortality is reduced. It is also important to mention that the side effects of statins may be benign, such as nausea, or severe, such as myoglobinuria, but this occurs extremely rarely.

## Figures and Tables

**Table 1:** Age-related diseases and their SASP profiles [6].

Disease	SASP Profile
Obesity	IL-1α, IL-6, IL-8
Diabetes	IL-1α, IL-6, IL-8
Hypertension	IL-1α, IL-6, IL-8
Atherosclerosis	IL-1α, IL-6, IL-8
Neurodegenerative diseases	p16, MMP i IL-6
Chronic obstructive pulmonary disease	IL-6, IL-8, MMP
Biliary cirrhosis	IL-6, IL-8, MMP
Cholangitis	IL-6, IL-8, MMP
Osteoarthritis	IL-6, IL-8, MMP

SASP, senescence-associated secretory phenotype; IL-1α, interleukin-1α; IL-6, interleukin-6; IL-8, interleukin-8; MMP, metalloproteinase.

**Table 2:** The anti-aging features of the Klotho protein [21].

Anti-Aging Features of the Klotho Protein
Anti-inflammatory effect
Protection against oxidative stress
Anti-aging of endothelial cells
Prevention of vascular calcification
Anti-fibrotic properties
Suppression of insulin/IGF-1 signaling pathway
Regulation of mineral metabolism

IGF-1, insulin-like growth factor-1.

## Data Availability

The data used in this article were sourced from materials mentioned in the References section.

## References

[1] Zhang L., Pitcher L.E., Yousefzadeh M.J., Niedernhofer L.J., Robbins P.D., Zhu Y. (2022). Cellular senescence: A key therapeutic target in aging and diseases. J. Clin. Investig..

[2] Docherty M.H., O’Sullivan E.D., Bonventre J.V., Ferenbach D.A. (2019). Cellular Senescence in the Kidney. J. Am. Soc. Nephrol..

[3] Shmulevich R., Krizhanovsky V. (2021). Cell Senescence, DNA Damage, and Metabolism. Antioxid. Redox Signal..

[4] Khalil R., Diab-Assaf M., Lemaitre J.M. (2023). Emerging Therapeutic Approaches to Target the Dark Side of Senescent Cells: New Hopes to Treat Aging as a Disease and to Delay Age-Related Pathologies. Cells.

[5] Cai H., Liu Y., Men H., Zheng Y. (2021). Protective Mechanism of Humanin Against Oxidative Stress in Aging-Related Cardiovascular Diseases. Front. Endocrinol..

[6] Liguori I., Russo G., Curcio F., Bulli G., Aran L., Della-Morte D., Gargiulo G., Testa G., Cacciatore F., Bonaduce D. (2018). Oxidative stress, aging, and diseases. Clin. Interv. Aging.

[7] Cabello-Verrugio C., Simon F., Trollet C., Santibañez J.F. (2017). Oxidative Stress in Disease and Aging: Mechanisms and Therapies 2016. Oxid. Med. Cell. Longev..

[8] Zhang H., Davies K.J.A., Forman H.J. (2015). Oxidative stress response and Nrf2 signaling in aging. Free Radic. Biol. Med..

[9] Yu C., Xiao J.H. (2021). The Keap1-Nrf2 System: A Mediator between Oxidative Stress and Aging. Oxid. Med. Cell. Longev..

[10] Mehdi M.M., Solanki P., Singh P. (2021). Oxidative stress, antioxidants, hormesis and calorie restriction: The current perspective in the biology of aging. Arch. Gerontol. Geriatr..

[11] Luo J., Mills K., le Cessie S., Noordam R., van Heemst D. (2020). Ageing, age-related diseases and oxidative stress: What to do next?. Ageing Res. Rev..

[12] Sarniak A., Lipińska J., Tytman K., Lipińska S. (2016). Endogenous mechanisms of reactive oxygen species (ROS) generation. Postepy Hig. Med. Dosw..

[13] Ray P.D., Huang B.W., Tsuji Y. (2012). Reactive oxygen species (ROS) homeostasis and redox regulation in cellular signaling. Cell. Signal..

[14] Kudryavtseva A.V., Krasnov G.S., Dmitriev A.A., Alekseev B.Y., Kardymon O.L., Sadritdinova A.F., Fedorova M.S., Pokrovsky A.V., Melnikova N.V., Kaprin A.D. (2016). Mitochondrial dysfunction and oxidative stress in aging and cancer. Oncotarget.

[15] El Assar M., Angulo J., Rodríguez-Mañas L. (2013). Oxidative stress and vascular inflammation in aging. Free Radic. Biol. Med..

[16] Shimizu I., Minamino T. (2019). Cellular senescence in cardiac diseases. J. Cardiol..

[17] Shafi O. (2020). Switching of vascular cells towards atherogenesis, and other factors contributing to atherosclerosis: A systematic review. Thromb. J..

[18] Jabarpour M., Rashtchizadeh N., Argani H., Ghorbanihaghjo A., Ranjbarzadhag M., Sanajou D., Panah F., Alirezaei A. (2019). The impact of dyslipidemia and oxidative stress on vasoactive mediators in patients with renal dysfunction. Int. Urol. Nephrol..

[19] Mok Y., Ballew S.H., Matsushita K. (2021). Chronic kidney disease measures for cardiovascular risk prediction. Atherosclerosis.

[20] Six I., Flissi N., Lenglet G., Louvet L., Kamel S., Gallet M., Massy Z.A., Liabeuf S. (2020). Uremic Toxins and Vascular Dysfunction. Toxins.

[21] Stenvinkel P., Larsson T.E. (2013). Chronic kidney disease: A clinical model of premature aging. Am. J. Kidney Dis..

[22] Ungvari Z., Tarantini S., Donato A.J., Galvan V., Csiszar A. (2018). Mechanisms of Vascular Aging. Circ. Res..

[23] Dai L., Qureshi A.R., Witasp A., Lindholm B., Stenvinkel P. (2019). Early Vascular Ageing and Cellular Senescence in Chronic Kidney Disease. Comput. Struct. Biotechnol. J..

[24] Carracedo J., Alique M., Vida C., Bodega G., Ceprián N., Morales E., Praga M., de Sequera P., Ramírez R. (2020). Mechanisms of Cardiovascular Disorders in Patients with Chronic Kidney Disease: A Process Related to Accelerated Senescence. Front. Cell Dev. Biol..

[25] Jebari-Benslaiman S., Galicia-García U., Larrea-Sebal A., Olaetxea J.R., Alloza I., Vandenbroeck K., Benito-Vicente A., Martín C. (2022). Pathophysiology of Atherosclerosis. Int. J. Mol. Sci..

[26] Bi X., Du C., Wang X., Wang X.Y., Han W., Wang Y., Qiao Y., Zhu Y., Ran L., Liu Y. (2021). Mitochondrial Damage-Induced Innate Immune Activation in Vascular Smooth Muscle Cells Promotes Chronic Kidney Disease-Associated Plaque Vulnerability. Adv. Sci..

[27] Düsing P., Zietzer A., Goody P.R., Hosen M.R., Kurts C., Nickenig G., Jansen F. (2021). Vascular pathologies in chronic kidney disease: Pathophysiological mechanisms and novel therapeutic approaches. J. Mol. Med..

[28] Bourrier M., Ferguson T.W., Embil J.M., Rigatto C., Komenda P., Tangri N. (2020). Peripheral Artery Disease: Its Adverse Consequences With and Without CKD. Am. J. Kidney Dis..

[29] Speer T., Ridker P.M., von Eckardstein A., Schunk S.J., Fliser D. (2021). Lipoproteins in chronic kidney disease: From bench to bedside. Eur. Heart J..

[30] Simeoni M., Borrelli S., Garofalo C., Fuiano G., Esposito C., Comi A., Provenzano M. (2021). Atherosclerotic-nephropathy: An updated narrative review. J. Nephrol..

[31] Zhou C., Shi Z., Ouyang N., Ruan X. (2021). Hyperphosphatemia and Cardiovascular Disease. Front. Cell Dev. Biol..

[32] Katsiki N., Mikhailidis D.P., Banach M. (2018). Leptin, cardiovascular diseases and type 2 diabetes mellitus. Acta Pharmacol. Sin..

[33] Singh A., Tandon S., Tandon C. (2021). An update on vascular calcification and potential therapeutics. Mol. Biol. Rep..

[34] Sevinc C., Yilmaz G., Ustundag S. (2021). The relationship between calcification inhibitor levels in chronic kidney disease and the development of atherosclerosis. Ren. Fail..

[35] Lv J.C., Zhang L.X., Liu B.C., Lan H.Y., Lv L.L. (2019). Prevalence and Disease Burden of Chronic Kidney Disease. Renal Fibrosis: Mechanisms and Therapies. Advances in Experimental Medicine and Biology.

[36] Wang J., Chen Z., Qiu Y., Wu L., Wang H., Wu L., Zhao L., Xie D. (2022). Statins Have an Anti-Inflammation in CKD Patients: A Meta-Analysis of Randomized Trials. BioMed Res. Int..

[37] Istvan E.S., Deisenhofer J. (2001). Structural mechanism for statin inhibition of HMG-CoA reductase. Science.

[38] Gunde-Cimerman N., Cimerman A. (1995). Pleurotus fruiting bodies contain the inhibitor of 3-hydroxy-3-methylglutaryl-coenzyme A reductase-lovastatin. Exp Mycol..

[39] Takemoto M., Liao J.K. (2001). Pleiotropic effects of 3-hydroxy-3-methylglutaryl coenzyme a reductase inhibitors. Arterioscler. Thromb. Vasc. Biol..

[40] García-Fernández-Bravo I., Torres-Do-Rego A., López-Farré A., Galeano-Valle F., Demelo-Rodriguez P., Alvarez-Sala-Walther L.A. (2022). Undertreatment or Overtreatment With Statins: Where Are We?. Front. Cardiovasc. Med..

[41] Agarwal R. (2007). Effects of statins on renal function. Mayo Clin. Proc..

[42] Turner A.J., Hick P.E. (1975). Inhibition of aldehyde reductase by acidic metabolites of the biogenic amines. Biochem. Pharmacol..

[43] Iseki K., Yamazato M., Tozawa M., Takishita S. (2002). Hypocholesterolemia is a significant predictor of death in a cohort of chronic hemodialysis patients. Kidney Int..

[44] Janda S., Young A., Fitzgerald J.M., Etminan M., Swiston J. (2010). The effect of statins on mortality from severe infections and sepsis: A systematic review and meta-analysis. J. Crit. Care.

[45] Tleyjeh I.M., Kashour T., Hakim F.A., Zimmerman V.A., Erwin P.J., Sutton A.J., Ibrahim T. (2009). Statins for the prevention and treatment of infections: A systematic review and meta-analysis. Arch. Intern. Med..

[46] Matsushita K., Sang Y., Ballew S.H., Astor B.C., Hoogeveen R.C., Solomon S.D., Ballantyne C.M., Woodward M., Coresh J. (2014). Cardiac and kidney markers for cardiovascular prediction in individuals with chronic kidney disease: The Atherosclerosis Risk in Communities study. Arterioscler. Thromb. Vasc. Biol..

[47] Esmeijer K., Dekkers O.M., de Fijter J.W., Dekker F.W., Hoogeveen E.K. (2019). Effect of different types of statins on kidney function decline and proteinuria: A network meta-analysis. Sci Rep..

[48] Sikora E., Bielak-Zmijewska A., Mosieniak G. (2021). A common signature of cellular senescence; does it exist?. Ageing Res. Rev..

[49] Roger L., Tomas F., Gire V. (2021). Mechanisms and Regulation of Cellular Senescence. Int. J. Mol. Sci..

[50] Zhang L., Pitcher L.E., Prahalad V., Niedernhofer L.J., Robbins P.D. (2023). Targeting cellular senescence with senotherapeutics: Senolytics and senomorphics. FEBS J..

[51] Lagoumtzi S.M., Chondrogianni N. (2021). Senolytics and senomorphics: Natural and synthetic therapeutics in the treatment of aging and chronic diseases. Free Radic. Biol. Med..

[52] Chaib S., Tchkonia T., Kirkland J.L. (2022). Cellular senescence and senolytics: The path to the clinic. Nat. Med..

[53] Birch J., Gil J. (2020). Senescence and the SASP: Many therapeutic avenues. Genes. Dev..

[54] Motoji Y., Fukazawa R., Matsui R., Abe Y., Uehara I., Watanabe M., Hashimoto Y., Miyagi Y., Nagi-Miura N., Tanaka N. (2022). Statins Show Anti-Atherosclerotic Effects by Improving Endothelial Cell Function in a Kawasaki Disease-like Vasculitis Mouse Model. Int. J. Mol. Sci..

[55] Martínez-González J., Badimon L. (2007). Influence of statin use on endothelial function: From bench to clinics. Curr. Pharm. Des..

[56] Girndt M., Seibert E. (2010). Premature cardiovascular disease in chronic renal failure (CRF): A model for an advanced ageing process. Exp. Gerontol..

[57] Hu C., Zhang X., Teng T., Ma Z.G., Tang Q.Z. (2022). Cellular Senescence in Cardiovascular Diseases: A Systematic Review. Aging Dis..

[58] Kousios A., Kouis P., Panayiotou A.G. (2016). Matrix Metalloproteinases and Subclinical Atherosclerosis in Chronic Kidney Disease: A Systematic Review. Int. J. Nephrol..

[59] Merino A., Buendia P., Martin-Malo A., Aljama P., Ramirez R., Carracedo J. (2011). Senescent CD14+CD16+ monocytes exhibit proinflammatory and proatherosclerotic activity. J. Immunol..

[60] Ho C.L.B., Chih H.J., Garimella P.S., Matsushita K., Jansen S., Reid C.M. (2021). Prevalence and risk factors of peripheral artery disease in a population with chronic kidney disease in Australia: A systematic review and meta-analysis. Nephrology.

[61] McCullough P.A., Sandberg K.R., Dumler F., Yanez J.E. (2004). Determinants of coronary vascular calcification in patients with chronic kidney disease and end-stage renal disease: A systematic review. J. Nephrol..

[62] Bahrami A., Bo S., Jamialahmadi T., Sahebkar A. (2020). Effects of 3-hydroxy-3-methylglutaryl coenzyme A reductase inhibitors on ageing: Molecular mechanisms. Ageing Res. Rev..

[63] Assmus B., Urbich C., Aicher A., Hofmann W.K., Haendeler J., Rössig L., Spyridopoulos I., Zeiher A.M., Dimmeler S. (2003). HMG-CoA reductase inhibitors reduce senescence and increase proliferation of endothelial progenitor cells via regulation of cell cycle regulatory genes. Circ. Res..

[64] Ushijima H., Onodera A. (2022). Selective Growth Suppressive Effect of Pravastatin on Senescent Human Lung Fibroblasts. Pharmazie.

[65] Liu S., Uppal H., Demaria M., Desprez P.Y., Campisi J., Kapahi P. (2015). Simvastatin suppresses breast cancer cell proliferation induced by senescent cells. Sci. Rep..

[66] Marcheggiani F., Cirilli I., Orlando P., Silvestri S., Vogelsang A., Knott A., Blatt T., Weise J.M., Tiano L. (2019). Modulation of Coenzyme Q10 content and oxidative status in human dermal fibroblasts using HMG-CoA reductase inhibitor over a broad range of concentrations. From mitohormesis to mitochondrial dysfunction and accelerated aging. Aging.

[67] Marcheggiani F., Kordes S., Cirilli I., Orlando P., Silvestri S., Vogelsang A., Möller N., Blatt T., Weise J.M., Damiani E. (2021). Anti-ageing effects of ubiquinone and ubiquinol in a senescence model of human dermal fibroblasts. Free Radic. Biol. Med..

